# Neighbourhood unemployment and other socio-demographic predictors of emergency hospitalisation for infectious intestinal disease in England: A longitudinal ecological study

**DOI:** 10.1016/j.jinf.2020.08.048

**Published:** 2020-11

**Authors:** Tanith C. Rose, Natalie L. Adams, Margaret Whitehead, Sophie Wickham, Sarah J. O'Brien, Jeremy Hawker, David C. Taylor-Robinson, Mara Violato, Benjamin Barr

**Affiliations:** a Department of Public Health, Policy and Systems, University of Liverpool, Waterhouse Building 2nd Floor Block F, Liverpool, UK; b School of Natural and Environmental Sciences, Newcastle University, Newcastle upon Tyne, UK; c National Infection Service, Public Health England, Birmingham, UK; d Health Economics Research Centre, University of Oxford, Oxford, UK

**Keywords:** Socioeconomic factors, Social class, Employment, Diarrhoea

## Abstract

**Background:**

Previous studies have observed that infectious intestinal disease (IID) related hospital admissions are higher in more deprived neighbourhoods. These studies have mainly focused on paediatric populations and are cross-sectional in nature. This study examines recent trends in emergency IID admission rates, and uses longitudinal methods to investigate the effects of unemployment (as a time varying measure of neighbourhood deprivation) and other socio-demographic characteristics on IID admissions for adults and children in England.

**Methods:**

A longitudinal ecological analysis was performed using Hospital Episode Statistics on emergency hospitalisations for IID, collected over the time period 2012–17 across England. Analysis was conducted at the neighbourhood (Lower-layer Super Output Area) level for three age groups (0–14; 15–64; 65+ years). Mixed-effect Poisson regression models were used to assess the relationship between trends in neighbourhood unemployment and emergency IID admission rates, whilst controlling for measures of primary and secondary care access, underlying morbidity and the ethnic composition of each neighbourhood.

**Results:**

From 2012–17, declining trends in emergency IID admission rates were observed for children and older adults overall, while rates increased for some sub-groups in the population. Each 1 percentage point increase in unemployment was associated with a 6.3, 2.4 and 4% increase in the rate of IID admissions per year for children [IRR=1.06, 95%CI 1.06–1.07], adults [IRR=1.02, 95%CI 1.02–1.03] and older adults [IRR=1.04, 95%CI 1.036–1.043], respectively. Increases in poor primary care access, the percentage of people from a Pakistani ethnic background, and the prevalence of long-term health problems, in a neighbourhood, were also associated with increases in IID admission rates.

**Conclusions:**

Increasing trends in neighbourhood deprivation, as measured by unemployment, were associated with increases in emergency IID admission rates for children and adults in England, despite controlling for measures of healthcare access, underlying morbidity and ethnicity. Research is needed to improve understanding of the mechanisms that explain these inequalities, so that effective policies can be developed to reduce the higher emergency IID admission rates experienced by more disadvantaged communities.

## Highlights

• We examined trends in infectious intestinal disease (IID) hospitalisations in England.
• Overall IID admission rates for children and older adults declined between 2012 & 2017.
• Increasing unemployment was associated with increasing IID admission rates.
• Healthcare access, underlying morbidity and ethnicity were also associated with IID rates.
• Policies should address inequalities in emergency IID hospitalisations.

## Background

Infectious intestinal disease (IID) is very common, with around 25% of the UK population experiencing an episode each year.^1^ Adverse consequences often include incapacitation and sickness absence,^2^ and hospitalisation in the most severe cases. In England, there were over 115,000 emergency admissions for IID in 2016/17.^3^ These admissions incur substantial costs for the National Health Service (NHS),^4^ as well as various costs for patients and families in terms of the disruption caused, work time lost and absences from school.^5^^,^^6^ Given that most hospitalisations for acute gastroenteritis are considered to be preventable with effective management,^7^ emergency hospital admissions for IID may represent expensive, yet potentially avoidable costs. Research is needed to examine changing trends in IID-related emergency admission rates, and to gain a better understanding of the factors which predict these admissions and the groups of people most likely to be affected.

Investigating the relationship between deprivation and hospitalisation rates for IID is of particular interest, since the association between deprivation and IID incidence appears to vary depending on the methods used to identify cases.^8^ One of our previous studies which analysed population-based survey data found that socially disadvantaged people in the UK have a lower risk of acquiring an IID in the community.^9^ In contrast, our study of calls to a free-phone NHS telephone advice service found higher call rates for gastrointestinal symptoms from more disadvantaged neighbourhoods.^10^ These seemingly conflicting findings could potentially relate to differences in healthcare utilisation^11^ and/or disease severity^12^ by social group.

Hospitalisation is in itself a severe consequence of having an IID, and a small number of UK-based studies have found that those living in more deprived areas compared to more affluent are more likely to be admitted to hospital with an IID^13^^,^^14^^,^^15^ or dehydration/gastroenteritis.^16^ These studies have, however, all been cross-sectional in nature and few have adequately controlled for potential confounding variables. For example, none has investigated the potential confounding effects of ethnicity on the relationship between deprivation and hospitalisation for IID.

Additionally, despite the importance of age as a risk factor for IID, very little is known about the modifying effect of age on the relationship between deprivation and emergency hospitalisation for IID. Previous cross-sectional studies have predominantly focused on children,^13^^,^^15^ or those of all ages combined,^16^ and only one study has presented results stratified by age.^14^ This latter study did not control for potential confounding variables, and data were collected over twenty years ago (1990–5) preceding recent clinical practice developments such as the introduction of the rotavirus vaccine into the UK childhood immunisation schedule in 2013. Therefore an up-to-date assessment is warranted.

To our knowledge, longitudinal methods have not been utilised to explore inequalities in hospitalisations for IID in a UK context, however these methods, whilst observational, provide better insights into temporal dynamics compared to cross-sectional analyses.^17^ In this study, we use longitudinal methods to investigate the relationship between trends in neighbourhood deprivation (measured using local unemployment prevalence data) and trends in emergency hospitalisations for IID, as well as examining other potential neighbourhood-level predictors of IID admissions, such as primary and secondary care access, ethnic composition and underlying morbidity. Analyses are stratified by age to explore the potential modifying effect of age on the relationships.

## Methods

### Study design and setting

We performed a longitudinal ecological analysis using data collected between 2012 and 2017, across Lower-layer Super Output Areas (LSOAs) in England. LSOAs (subsequently referred to as neighbourhoods) are small geographical areas used by the UK's Office for National Statistics (ONS), each containing a population of between 1000 and 3000 people.^18^ We analysed data from 2012 to 17 because covariate data were only available for this time period.

### Data sources and measures

We used Hospital Episode Statistics (HES) to derive our outcome: emergency hospital admissions for IID as a primary diagnosis (ICD-10 codes: A00–A09 intestinal infectious diseases, and K52.9 unspecified non-infective gastroenteritis and colitis). Emergency admissions and ONS population estimates were derived for each neighbourhood, per year from 2012 to 17, for three age groups (0–14; 15–64; 65+ years).^19^^,^^20^ The primary exposure of interest was the annual unemployment prevalence per neighbourhood, used as a time varying measure of area deprivation. This was measured as the percentage of people aged 16–64 years claiming Jobseeker's Allowance or Universal Credit principally for the reason of being unemployed. This definition differs from the definition of unemployment specified by the International Labour Organisation, which is not available at small neighbourhood levels.^21^ Our measure of unemployment, as described above, is the only measure of socioeconomic conditions that is available annually for small neighbourhoods in England. Other measures such as the Index of Multiple Deprivation (IMD) are only available for snapshots in time and these cannot be compared over time.^22^ Whilst our measure of unemployment directly relates to people of working age, in this analysis it is used as a general measure of trends in socioeconomic deprivation in each neighbourhood.

Several covariates were included in the analysis. Time-varying covariates related to primary care access included the number of general practitioners (GPs) per capita serving the population (derived from NHS Digital data); and the proportion of the population who would describe their experience of making an appointment as poor (derived from General Practice Patient Survey [GPPS] data). Previous studies have found that the GPPS measure of experience of making an appointment is strongly associated with measures relevant to patients’ self-reported access to services, i.e. the ability to get an appointment, appointment convenience, ease of telephone contact and helpfulness of receptionists.^23^

We also included a measure of secondary care access, which was the average road network distance to the nearest hospital with an Accident and Emergency (A&E) department. Data from the 2011 Census were used to derive time-invariant covariates relating to the proportion of different ethnic groups, and the age-specific prevalence of long-term health problems per neighbourhood. A full description of the measures and data sources can be found in the Supplementary file.

### Statistical analysis

We used mixed-effect Poisson regression models to investigate the association between annual changes in unemployment and changes in emergency IID hospitalisations between neighbourhoods, from 2012 to 17, whilst controlling for measures of primary and secondary care access, underlying morbidity and the ethnic composition of each neighbourhood. Numbers of emergency admissions were modelled using the log of the population as an ‘offset’ variable, indicating the maximum number of admissions that could have occurred. We also included in the model an annual time-trend term and a random intercept for each neighbourhood to account for the longitudinal nature of the data (see Supplementary file for full details of the statistical model). Analyses were conducted for three age groups (0–14; 15–64; 65+ years), using R (version 3.6.0) and Stata (version 14).

### Robustness tests

We repeated the analysis using a different definition of IID that excluded ICD-10 codes K52.9 and A09.9 (gastroenteritis and colitis of unspecified origin). This definition of IID was more specific, but was probably less sensitive, given that possible cases of IID were likely excluded. We also repeated our analyses using mixed-effect negative binomial models which would account for any overdispersion within the data. Additionally, using data collected over a longer time period (2005–17) we investigated the association between changes in unemployment and changes in emergency IID admission rates within neighbourhoods using fixed-effect Poisson models. These models include a fixed effect for each neighbourhood, and thereby indicate the relationship between unemployment trends within neighbourhoods and trends in IID admissions whilst controlling for all time-invariant differences between neighbourhoods.^24^

## Results

**Fig. 1:**
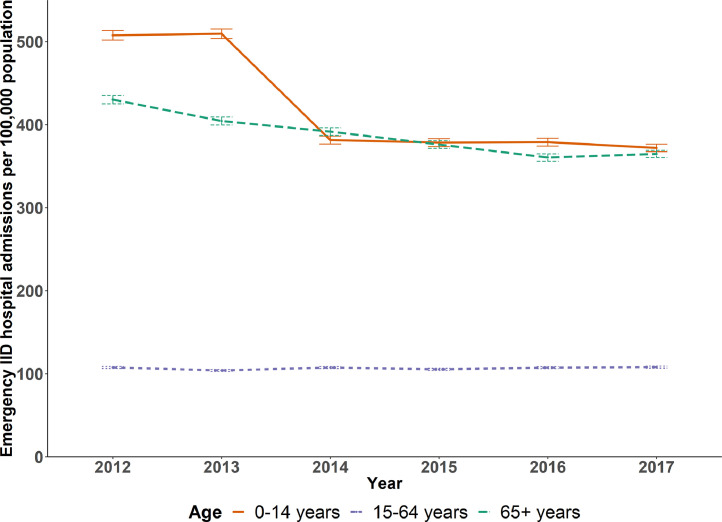
Emergency IID hospital admission rates for English neighbourhoods by age group, 2012–17.

In total, 32,829 English neighbourhoods had available data from 2012 to 17, giving a total sample size of 196,974 LSOA-years for each age group. Fig. 1 shows the trends in emergency IID admission rates for three age groups between 2012 and 2017.

Prior to 2014, emergency IID admission rates were highest amongst children, followed by adults aged 65+ years, and adults aged 15–64 years (Fig. 1). For children, there was a stepwise 25% reduction in admission rates between 2013 and 2014 coinciding with the introduction of the rotavirus vaccine and rates remained stable for other years. For older adults aged 65+ years there was a fairly constant decline in emergency IID admission rates over the time period from 2012 to 17 (a 15% reduction), whilst for adults aged 15–64 years the rate remained fairly constant at just over 100 per 100,000. Unemployment prevalence decreased from 3.7% in 2012 to 1.8% in 2015, and remained stable for other years (see Supplementary file for annual emergency admission rates, unemployment prevalence and other characteristics of English neighbourhoods).

Results from the regression analysis showed that increasing trends in neighbourhood unemployment were associated with increasing trends in emergency IID admission rates, for all age groups (Table 1, Table 2, Table 3, Table 4). In univariate analysis, every 1 percentage point increase in unemployment was associated with a 6.5, 8.5 and 9% increase in the rate of emergency IID admissions per year for children, adults and older adults, respectively.

**Table 1:** Univariate Poisson regression models showing the effect of change in unemployment on change in emergency IID admissions per 100,000 population, for English neighbourhoods, stratified by age group, 2012–2017.

	Children aged 0–14 years			Adults aged 15–64 years			Adults aged 65+ years		
	IRR	Lower 95% CI	Upper 95% CI	IRR	Lower 95% CI	Upper 95% CI	IRR	Lower 95% CI	Upper 95% CI
Working age population unemployed (%)	1.065	1.062	1.068	1.085	1.082	1.088	1.09	1.086	1.093

Models include random intercept for LSOA, and fixed effects for year (full model results are given in Supplementary file).

Models based on 196,974 observations.

CI = confidence interval; IID = infectious intestinal disease; IRR = incident rate ratio; LSOA = Lower-layer Super Output Area.

**Table 2:** Multivariable Poisson regression model for emergency IID admissions per 100,000 children aged 0–14 years, for English neighbourhoods, 2012–2017.

	IRR	p-value	Lower 95% CI	Upper 95% CI
Working age population unemployed (%)	1.063	<0.001	1.059	1.066
Prevalence long-term health problems, children aged 0–14 years (%)	1.042	<0.001	1.037	1.047
Population who would describe their experience of making a GP/nurse appointment as poor (%)	1.002	0.001	1.001	1.003
GPs per 1000 population	0.926	0.011	0.874	0.983
Travelling distance to hospital (km)	0.995	<0.001	0.994	0.996
Ethnic group: Black (%)	0.984	<0.001	0.983	0.986
Ethnic group: Chinese (%)	0.977	<0.001	0.969	0.986
Ethnic group: Bangladeshi (%)	0.991	<0.001	0.989	0.994
Ethnic group: Indian (%)	0.998	0.004	0.997	0.999
Ethnic group: Pakistani (%)	1.01	<0.001	1.009	1.011

Model includes random intercept for LSOA, and fixed effects for year (full model results are given in Supplementary file).

Model based on 196,974 observations.

CI = confidence interval; GP = general practitioner; IID = infectious intestinal disease; IRR = incident rate ratio; km = kilometre; LSOA = Lower-layer Super Output Area.

**Table 3:** Multivariable Poisson regression model for emergency IID admissions per 100,000 adults aged 15–64 years, for English neighbourhoods, 2012–2017.

	IRR	p-value	Lower 95% CI	Upper 95% CI
Working age population unemployed (%)	1.024	<0.001	1.021	1.028
Prevalence long-term health problems, adults aged 15–64 years (%)	1.041	<0.001	1.039	1.043
Population who would describe their experience of making a GP/nurse appointment as poor (%)	1.004	<0.001	1.003	1.005
GPs per 1000 population	1.106	<0.001	1.053	1.163
Travelling distance to hospital (km)	0.994	<0.001	0.993	0.995
Ethnic group: Black (%)	1	0.778	0.999	1.001
Ethnic group: Chinese (%)	0.987	<0.001	0.983	0.991
Ethnic group: Bangladeshi (%)	0.996	<0.001	0.995	0.998
Ethnic group: Indian (%)	1.003	<0.001	1.002	1.004
Ethnic group: Pakistani (%)	1.003	<0.001	1.003	1.004

Model includes random intercept for LSOA, and fixed effects for year (full model results are given in Supplementary file).

Model based on 196,974 observations.

CI = confidence interval; GP = general practitioner; IID = infectious intestinal disease; IRR = incident rate ratio; km = kilometre; LSOA = Lower-layer Super Output Area.

**Table 4:** Multivariable Poisson regression model for emergency IID admissions per 100,000 adults aged 65+ years, for English neighbourhoods, 2012–2017.

	IRR	p-value	Lower 95% CI	Upper 95% CI
Working age population unemployed (%)	1.04	<0.001	1.036	1.043
Prevalence long-term health problems, adults aged 65+ years (%)	1.014	<0.001	1.013	1.015
Population who would describe their experience of making a GP/nurse appointment as poor (%)	1.005	<0.001	1.004	1.006
GPs per 1000 population	1.015	0.603	0.959	1.074
Travelling distance to hospital (km)	0.99	<0.001	0.989	0.991
Ethnic group: Black (%)	1.003	<0.001	1.002	1.004
Ethnic group: Chinese (%)	1.025	<0.001	1.019	1.031
Ethnic group: Bangladeshi (%)	1.001	0.298	0.999	1.003
Ethnic group: Indian (%)	1	0.493	0.999	1.001
Ethnic group: Pakistani (%)	1.003	<0.001	1.002	1.004

Model includes random intercept for LSOA, and fixed effects for year (full model results are given in Supplementary file).

Model based on 196,974 observations.

CI = confidence interval; GP = general practitioner; IID = infectious intestinal disease; IRR = incident rate ratio; km = kilometre; LSOA = Lower-layer Super Output Area.

Following adjustment for measures of primary and secondary care access, underlying morbidity and ethnic composition, the magnitudes of these associations were attenuated but remained statistically significant (Table 2, Table 3, Table 4). Each 1 percentage point increase in unemployment was associated with a 6.3, 2.4 and 4% increase in the rate of emergency IID admissions per year for children [IRR=1.06, 95%CI 1.06 to 1.07], adults [IRR=1.02, 95%CI 1.02 to 1.03] and older adults [IRR=1.04, 95%CI 1.036 to 1.043], respectively. This is approximately equivalent to an additional 2356, 922 and 1464 emergency admissions per year for children, adults and older adults, respectively, in England, for every 1 percentage point increase in unemployment.

For all age groups, the increasing prevalence of long-term health problems was associated with increasing emergency IID admission rates. The effects of ethnicity appeared to vary by age group, with one exception: as the percentage of people from a Pakistani ethnic background increased in a neighbourhood, emergency IID admission rates for all age groups increased.

In terms of primary care access, increasing trends in the percentage of the population who would describe their experience of making a GP/nurse appointment as poor, were associated with increasing trends in emergency IID admission rates for all age groups. Increasing trends in GPs per capita were associated with decreasing trends in emergency IID admission rates for children, and increasing trends for adults aged 15–64 years. In terms of secondary care access, neighbourhoods situated closer to hospitals with A&E departments were associated with higher emergency IID admission rates for all age groups.

### Robustness tests

We found similar results when repeating the analyses using mixed-effect negative binomial models and using a more specific definition of IID (Supplementary file). Fixed-effect Poisson models, which control for all time-invariant unobserved differences between neighbourhoods, also showed that increasing trends in neighbourhood unemployment were associated with increasing trends in emergency IID admission rates over a longer time period (2005–17), for all age groups (Supplementary file). These models however indicated a slightly smaller effect indicating that each 1 percentage point increase in unemployment was associated with a 1.7, 1.8 and 1.0% increase in the rate of emergency IID admissions per year for children [IRR=1.017, 95%CI 1.013 to 1.021], adults [IRR=1.018, 95%CI 1.014 to 1.022] and older adults [IRR=1.010, 95%CI 1.006 to 1.014], respectively.

## Discussion

This longitudinal analysis of English neighbourhoods found that increasing trends in neighbourhood unemployment were associated with increasing trends in emergency infectious intestinal disease admission rates, for children, adults and older adults, despite controlling for factors such as ethnicity, underlying morbidity, travelling distance to hospital and experience of making a primary care appointment. Adjusting for these covariates attenuated much of the association between trends in neighbourhood unemployment and trends in emergency IID admission rates for adults, but not for children. Robustness tests using fixed-effect models produced effect estimates that were smaller in magnitude, suggesting that some of the association from the main analysis could be due to residual confounding, but that there appears to be an association between trends in unemployment and emergency IID admission rates even when all time-invariant unobserved confounders are controlled for.

Some^13^, ^14^, ^15^, ^16^ but not all^25^ previous UK-based studies have observed social gradients in IID-related hospital admissions. The majority of these studies have focused on paediatric populations^13^^,^^15^^,^^25^ or those of all ages combined,^16^ and only one has investigated inequalities in IID admissions amongst adults specifically.^14^ This previous study, conducted using data from 1990 to 95, included admissions where IID was recorded as a primary or secondary diagnosis, and found inequalities in admission rates between the most and least deprived neighbourhoods for both children and adults in the West Midlands region of England.^14^ Our study adds to the evidence base by using the most recent data available across England and robust longitudinal methods, to demonstrate a relationship between socioeconomic trends and trends in emergency IID admission rates within neighbourhoods over time.

Socially disadvantaged people tend to present to healthcare services more often, because they experience a greater burden of disease,^26^ however in terms of IID, some UK-based evidence suggests that there may not be significant inequalities in IID incidence in the community.^9^ This points to alternative explanations for the social gradients in emergency IID admission rates that have been observed – for example inequalities in disease severity. Our analysis of the UK-based IID2 study found that disadvantaged children and adults tend to experience greater levels of IID severity in terms of the symptoms they experience and the duration of these symptoms.^12^ Other studies analysing survey data have found that IID severity is strongly predictive of GP presentation for IID, with cases experiencing severe illness having 12 times the odds of presenting to their GP compared to those with mild illness.^11^ Inequalities in disease severity may therefore contribute to a greater need for healthcare amongst more disadvantaged communities. Other hypothetical explanations for our findings could relate to ‘pro-poor’ biases in GP referral practices or decisions to admit, or differences in decision thresholds for seeking medical advice between socioeconomic groups. Further research is needed, especially qualitative research,^27^ to increase understanding of the mechanisms that explain the inequalities observed.

Our study also adds to the evidence base by revealing insights about other predictive factors of emergency IID admissions. For example, the age-specific prevalence of long-term health problems was included in our models as a measure of underlying morbidity and was independently associated with increasing emergency IID admission rates for all age groups. There are several common chronic conditions that can result in secondary immunodeficiency or require immune suppressing treatments/medications or acid-suppression medications, and as such these conditions may increase susceptibility to and severity of IID.^28^^,^^29^ As would be expected, the prevalence of long-term health problems for adults compared to children was more closely correlated with unemployment prevalence (see Supplementary file, correlation matrix), and thus may have explained more of the association between trends in unemployment and IID admissions amongst adults.

We also explored whether measures of primary care access were associated with IID admission rates, since in most cases hospitalisation for IID can be avoided through early intervention and effective management.^7^ Our results suggest that emergency IID admission rates for all age groups increase, when people in general report poor experiences when making primary care appointments. Previous studies have also found associations between poor experiences of making appointments and higher rates of all-cause A&E visits and emergency admissions.^30^ In other studies, GP continuity has been associated with lower rates of unplanned hospital admissions for dehydration and gastroenteritis.^16^ Strategies to reduce emergency IID admissions may therefore wish to address modifiable primary care factors such as these. On the other hand, we found that increasing trends in GPs per capita were associated with decreasing trends in emergency IID admission rates for children, but increasing trends for adults aged 15–64 years. Mixed findings have also been observed by studies that have investigated the association between GP supply and all-cause emergency admissions.^31^

In terms of secondary care access, our results suggest that emergency IID admission rates for all age groups are higher when people have a shorter travelling distance to get to a hospital with an A&E department. Previous studies have found similar relationships between closer residential proximity to hospitals and higher all-cause or disease-specific emergency admission rates.^16^^,^^32^^,^^33^ Some studies have expressed uncertainty about whether residents who live in closer proximity to A&E services have higher emergency admission rates in general because they find it easier to access these services or because of wider factors relating to access and quality of primary care.^34^^,^^35^ In this analysis, however, we were able to observe an association between distance to hospital and IID admission rates whilst controlling for measures of primary care access.

Finally, age appeared to modify the relationship between ethnicity and emergency IID admission rates. Neighbourhoods that had a greater proportion of residents from Black, Chinese, Bangladeshi and Indian backgrounds, had lower admission rates for children, however the direction of these associations tended to be reversed for older adults. There was one exception however: as the percentage of people from a Pakistani ethnic background increased in a neighbourhood, emergency IID admission rates for all age groups increased. A previous UK-based study, which analysed those of all ages combined, found that Pakistani communities had an increased risk of laboratory-confirmed Campylobacter infection compared to White communities, whilst Black and Indian communities had a decreased risk.^36^ Further research is needed to shed light on the reasons for these findings.

### Strengths and limitations

Our analysis shows for the first time in the UK, that trends over time in neighbourhood unemployment are associated with trends in emergency IID admission rates for children and adults. We performed a comprehensive analysis of all emergency hospital admissions for IID in England over a six year period, and as such it can be assumed that our results are generalisable to the English population. A number of robustness tests confirmed our results, using different types of models and a more specific definition of IID.

There are limitations, however, that should be considered when interpreting the results. Firstly, information on repeat admissions by the same individual and hospital identifiers were not available within the dataset, which precluded the investigation of clustering at the individual and hospital level. It is not known to what extent these factors may have influenced the results of the study. However our previous studies investigating inequalities in the risks and consequences of IID using individual-level data have found that inequalities, where present, persist even after accounting for recurrent IID.^9^ ^12^

In terms of study design, methodological limitations of ecological studies can include ecological bias whereby associations present at the group-level are not apparent at the individual-level, possibly due to unmeasured confounding or measurement error.^37^ Nonetheless, data were aggregated to relatively small areas (LSOAs containing 1000 to 3000 people) which likely limited the effects of ecological bias. Additionally, because ecological studies are able to capture risk factors and exposures that operate at the community-level,^38^ it could be argued that ecological studies are in fact more appropriate for the study of infectious diseases compared to individual-level studies. Individual-level studies may however be best placed to examine some of the associations observed in this study in greater depth.

In conclusion, the findings from this study indicate that emergency IID hospitalisation (a severe consequence of having an IID) disproportionately affects disadvantaged communities. Given that most hospital admissions for IID are considered to be preventable, the findings from this study are particularly disconcerting. Unplanned admission to hospital can be distressing and disruptive for patients and families,^39^^,^^40^ and can incur costs such as loss of employment opportunities/income which might have more damaging effects for those in lower social positions who have less financial cushioning.^41^ Reducing emergency IID hospital admission rates experienced by the most disadvantaged to levels experienced by the least, should be an important goal for any levelling-up policy or intervention designed to improve equity in health.^42^ Policies to improve primary care access have potential to reduce emergency IID admission rates, however the results of this study suggest that due consideration should be afforded to ensuring that these policies adequately address inequalities in IID admissions.

## Funding

This report is independent research part funded by: 1) the National Institute for Health Research Health Protection Research Unit (NIHR HPRU) in Gastrointestinal Infections at the University of Liverpool in partnership with Public Health England (PHE), in collaboration with University of East Anglia, University of Oxford and the Quadram Institute; and 2) the National Institute for Health Research Applied Research Collaboration North West Coast (ARC NWC). The views expressed in this publication are those of the author(s) and not necessarily those of the National Institute for Health Research, the Department of Health and Social Care, the NHS or PHE. DTR is funded by the MRC on a Clinician Scientist Fellowship (MR/P008577/1). SW is funded by a Wellcome Trust Society and Ethics Research Fellowship (200,335/Z/15/Z).

## Contributions

TR, NA, MW, SO, JH, MV, DTR and BB conceptualised the study. TR analysed the data and drafted the manuscript with support from BB, DTR, MV and JH. TR, NA, MW, SW, SO, JH, MV, DTR and BB interpreted the data and revised the manuscript. All authors approved the submitted version of the manuscript.

## Ethics

No ethical approval was required for this study, as it involved the use of anonymous aggregate secondary health service data and openly available data.

## Declaration of Competing Interest

None

## Appendix Supplementary materials

Image, application 1
