# Supplementary material for: Neighbourhood unemployment and other socio-demographic predictors of emergency hospitalisation for infectious intestinal disease in England: A longitudinal ecological study
Source: J Infect. 2020 Nov;81(5):736–42. doi: 10.1016/j.jinf.2020.08.048 (PMC7649336; doi:10.1016/j.jinf.2020.08.048)
Supplement: Supplementary file 1 [file mmc1.pdf]

Supplementary file

Data sources and measures

The following variables were included in the analysis:

Variable	Details
Unemployment	Unemployment prevalence per year was calculated using claimant data provided by the ONS. Unemployment was measured as the percentage of people aged 16–64 years claiming Jobseeker's Allowance or Universal Credit principally for the reason of being unemployed.
Infectious intestinal disease (IID) emergency hospital admissions	Emergency admissions for IID were defined using ICD-10 codes: A00–A09 (intestinal infectious diseases) and K52.9 (unspecified non-infective gastroenteritis and colitis). Total numbers of IID emergency admissions per calendar year, for three age groups (0–14; 15–64; 65+ years) were calculated using Hospital Episode Statistics (HES). Continuous inpatient (CIP) spells were used to define emergency admissions.
Number of general practitioners (GPs) per capita serving the population	Weighted averages of the number of full-time employed GPs per 1000 population were calculated using annual data provided by NHS Digital on the number of GPs and patients registered per general practice per LSOA.
Experience of making a GP or nurse appointment	General Practice Patient Survey (GPPS) data were used to derive a measure for the proportion of the population who would describe their experience of making an appointment the last time they wanted to see or speak to a GP or nurse from their surgery, as fairly poor or very poor. Weighted averages of the indicator per LSOA and year were calculated using annual data provided by NHS Digital on the total number of patients registered per general practice per LSOA.
Ethnic group	The proportion of the population who classify their own perceived ethnic group and cultural background as: Black / African / Caribbean / Black British / any other Black / African / Caribbean background; Asian / Asian British – Indian; Asian / Asian British – Pakistani; Asian / Asian British – Bangladeshi; Asian / Asian British – Chinese. The ONS provide data about the ethnic group of the usual resident population of England as at census day, 27 March 2011.
Prevalence of long-term health problems	The proportion of the population who report having a long-term health problem or disability was calculated for three age groups (0–14; 15–64; 65+ years) using census data. The 2011 Census defines a long-term health problem or disability as that which limits a person's day-to-day activities, and has lasted or is expected to last at least 12 months, including problems that are related to old age.

Distance to the nearest hospital	The Consumer Data Research Centre provided data per LSOA on the average road network distance to the nearest hospital with an Accident and Emergency (A&E) department. Road network distances in kilometres were calculated by deriving the fastest route by car to travel from each postcode within an LSOA to the nearest health service.

Statistical model

Equation for multivariable Poisson regression model with random coefficients

$$\log E(Y_{ij}|u_i) = \alpha + u_i + \beta_1 T_{ij} + \beta_2 Health_{ij} + \beta_3 EthnicityBlack_{ij} + \beta_4 EthnicityIndian_{ij} + \beta_5 EthnicityPakistani_{ij} + \beta_6 EthnicityBangladeshi_{ij} + \beta_7 EthnicityChinese_{ij} + \beta_8 Unemployment_{ij} + \beta_9 Appointment_{ij} + \beta_{10} GPperCapita_{ij} + \beta_{11} Distance_{ij} + log(population_{ij})$$

Where Y_{ij} is the number of IID emergency admissions for LSOA *i* at year *j* and u_i is the random effect for LSOA *i*. *T* is an annual time-trend term. *Health* is the percent of the population who report having a long-term health problem or disability. *EthnicityBlack* is the percent of the population who classify their ethnic group as: Black / African / Caribbean / Black British / Black Other. *EthnicityIndian* is the percent of the population who classify their ethnic group as: Asian / Asian British – Indian. *EthnicityPakistani* is the percent of the population who classify their ethnic group as: Asian / Asian British – Pakistani. *EthnicityBangladeshi* is the percent of the population who classify their ethnic group as: Asian / Asian British – Bangladeshi. *EthnicityChinese* is the percent of the population who classify their ethnic group as: Asian / Asian British – Chinese. *Unemployment* is the percent of the working age population (aged 16–64 years) claiming Jobseeker's Allowance or Universal Credit principally for the reason of being unemployed. *Appointment* is the percent of the population who would describe their experience of making a GP/nurse appointment as fairly poor or very poor. *GPperCapita* is the number of full-time employed GPs per 1000 population. *Distance* is the average road network distance in kilometres to the nearest hospital with an A&E department. *Population* is an offset variable with a parameter estimate constrained to 1.

Characteristics of English neighbourhoods, 2012–17

	Mean (SD)
Working age population unemployed (%)	2.41 (2.28)
Prevalence long-term health problems children aged 0–14 years (%)	3.57 (1.62)
Prevalence long-term health problems adults aged 15–64 years (%)	12.52 (4.70)
Prevalence long-term health problems adults aged 65+ years (%)	50.81 (10.71)
Population who would describe their experience of making a GP/nurse appointment as poor (%)	11.18 (5.74)
GPs per 1000 population	0.51 (0.10)
Travelling distance to hospital with A&E (km)	6.61 (4.98)
Ethnic group: Black (%)	3.29 (6.64)
Ethnic group: Chinese (%)	0.68 (1.23)
Ethnic group: Bangladeshi (%)	0.76 (3.27)
Ethnic group: Indian (%)	2.50 (5.97)
Ethnic group: Pakistani (%)	1.92 (6.32)

Data based on 32,829 English neighbourhoods
A&E = Accident and Emergency department; GP = general practitioner; GPPS = General Practice Patient Survey; km = kilometres; SD = standard deviation

Emergency IID hospital admissions per 100,000 population per year

	Children aged 0–14 years			Adults aged 15–64 years			Adults aged 65+ years		
Year	Mean	Lower 95% CI	Upper 95% CI	Mean	Lower 95% CI	Upper 95% CI	Mean	Lower 95% CI	Upper 95% CI
2012	507.8	502.0	513.6	107.7	106.5	108.9	430.3	425.2	435.4
2013	509.7	503.9	515.5	104.1	102.9	105.3	404.6	399.7	409.4
2014	381.6	376.8	386.4	107.6	106.4	108.8	391.8	387.0	396.5
2015	378.6	373.9	383.3	105.4	104.2	106.6	376.2	371.6	380.8
2016	379.1	374.4	383.8	107.5	106.3	108.7	360.6	356.2	365.0
2017	372.2	367.6	376.7	108.1	106.9	109.3	364.9	360.5	369.2

Data based on 32,829 English neighbourhoods
CI = confidence interval; IID = infectious intestinal disease

Annual unemployment prevalence – proportion of people aged 16–64 years claiming Jobseeker's Allowance or Universal Credit principally for the reason of being unemployed, for English neighbourhoods, 2012–17

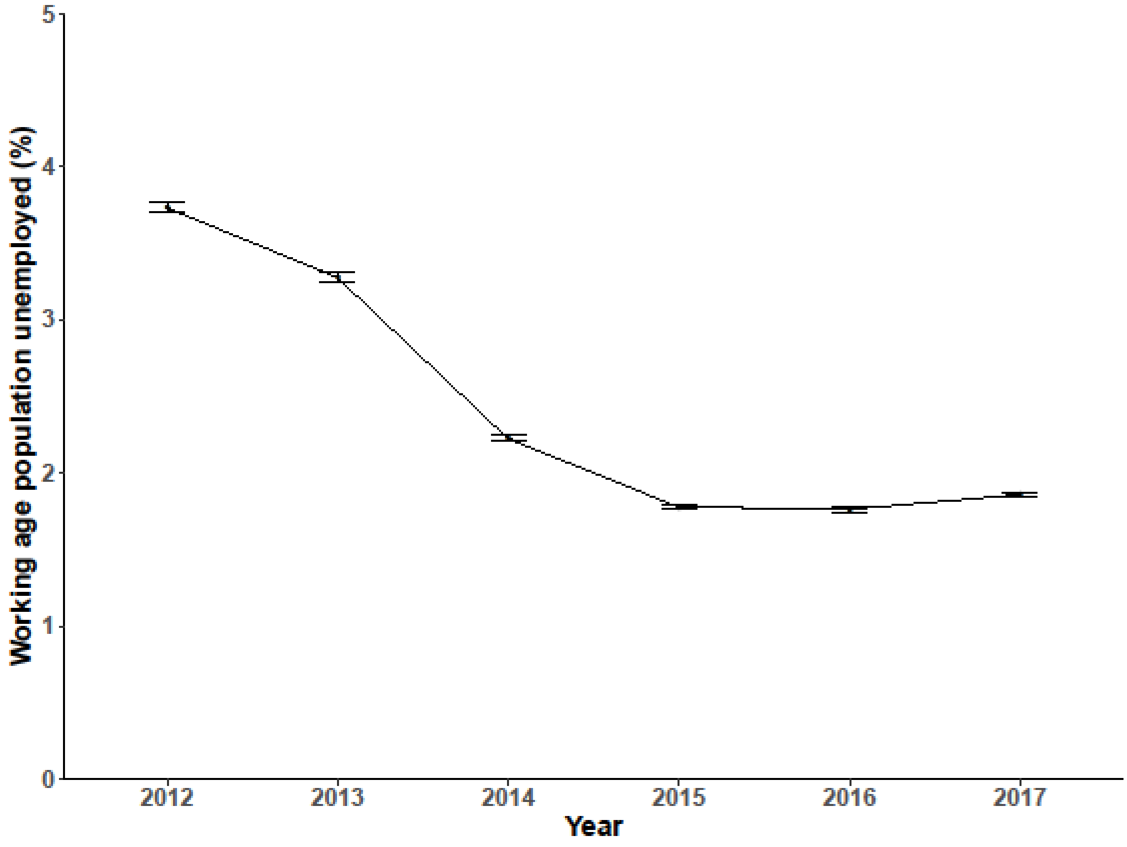

Correlation matrix of covariates, 2012-17

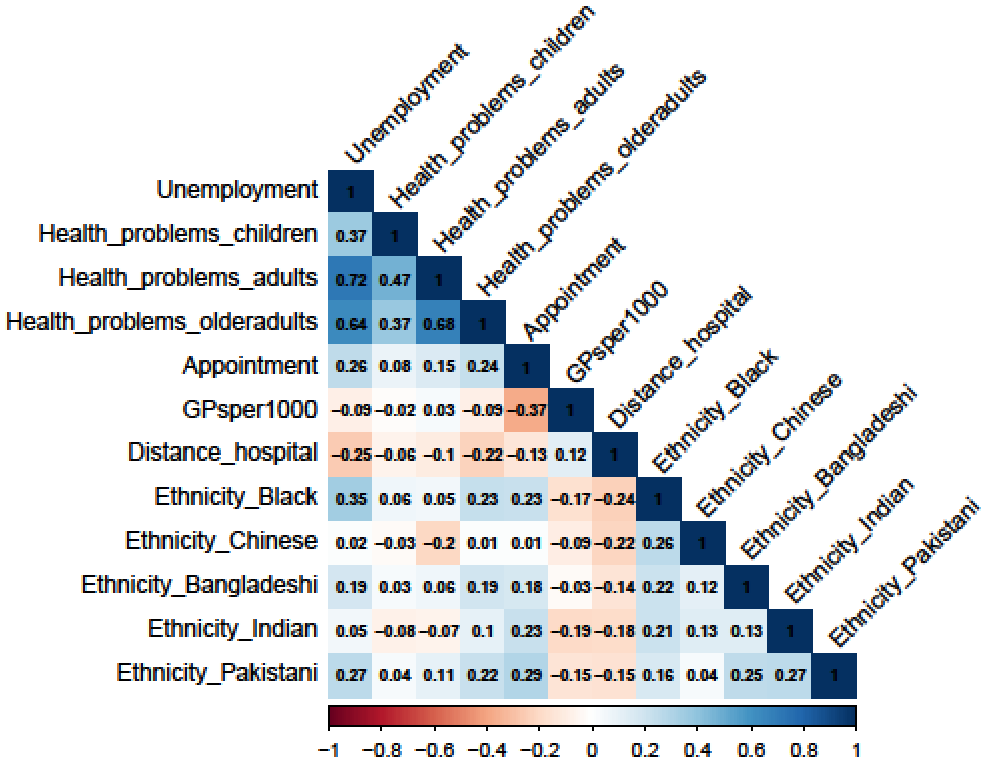

Emergency IID hospital admission rates for English neighbourhoods by age group, 2005–17

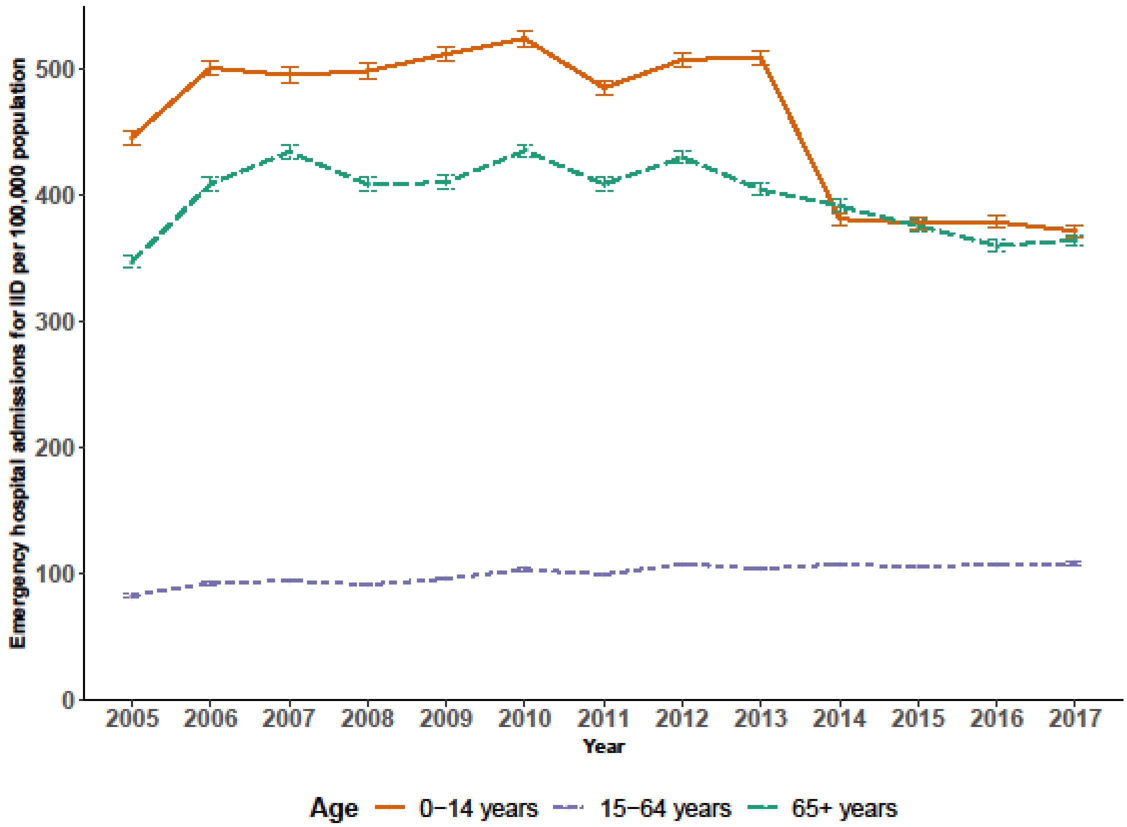

Models – main analysis

Age group: 0-14 years

Mixed-effect Poisson regression model showing the effect of change in unemployment on change in emergency IID admissions per 100,000 children aged 0–14 years, for English neighbourhoods, 2012–2017

	IRR	St.Err.	t-value	p-value	[95% Conf	Interval]	Sig
Working age population unemployed (%)	1.065	0.002	42.99	<0.001	1.062	1.068	***
2012.year	1.000	.	.	.	.	.	
2013.year	1.037	0.007	5.19	<0.001	1.023	1.051	***
2014.year	0.843	0.007	-20.98	<0.001	0.829	0.856	***
2015.year	0.865	0.007	-17.16	<0.001	0.851	0.879	***
2016.year	0.866	0.007	-16.84	<0.001	0.852	0.881	***
2017.year	0.844	0.007	-20.05	<0.001	0.830	0.858	***
Constant	0.004	0.000	-648.34	<0.001	0.004	0.004	***
Constant	-1.307	0.071	.b	.b	-1.446	-1.168	

Mean dependent var	1.249	SD dependent var	1.534
Number of obs	196974.000	Chi-square	2343070.477
Prob > chi2	0.000	Akaike crit. (AIC)	565089.949

**** p<0.01, ** p<0.05, * p<0.1*

Multivariable mixed-effect Poisson regression model for emergency IID admissions per 100,000 children aged 0–14 years, for English neighbourhoods, 2012–2017

	IRR	St.Err.	t-value	p-value	[95% Conf	Interval]	Sig
Working age population unemployed (%)	1.063	0.002	35.85	<0.001	1.059	1.066	***
Prevalence long-term health problems, children aged 0-14 years (%)	1.042	0.002	17.47	<0.001	1.037	1.047	***
Population who would describe their experience of making a GP/nurse appointment as poor (%)	1.002	0.001	3.40	0.001	1.001	1.003	***
GPs per 1000 population	0.926	0.028	-2.55	0.011	0.874	0.983	**
Travelling distance to hospital (km)	0.995	0.001	-6.73	<0.001	0.994	0.996	***
Ethnic group: Black (%)	0.984	0.001	-25.86	<0.001	0.983	0.986	***
Ethnic group: Chinese (%)	0.977	0.004	-5.34	<0.001	0.969	0.986	***
Ethnic group: Bangladeshi (%)	0.991	0.001	-6.80	<0.001	0.989	0.994	***
Ethnic group: Indian (%)	0.998	0.001	-2.85	0.004	0.997	0.999	***
Ethnic group: Pakistani (%)	1.010	0.001	18.02	<0.001	1.009	1.011	***
2012.year	1.000	.	.	.	.	.	
2013.year	1.032	0.007	4.47	<0.001	1.018	1.047	***
2014.year	0.835	0.007	-21.16	<0.001	0.821	0.849	***
2015.year	0.853	0.008	-17.81	<0.001	0.838	0.868	***
2016.year	0.853	0.008	-17.69	<0.001	0.838	0.868	***
2017.year	0.829	0.007	-20.77	<0.001	0.815	0.844	***
Constant	0.004	0.000	-260.72	<0.001	0.004	0.004	***
Constant	-1.399	0.075	.b	.b	-1.547	-1.252	

Mean dependent var	1.249	SD dependent var	1.534
Number of obs	196974.000	Chi-square	2497609.133
Prob > chi2	0.000	Akaike crit. (AIC)	563368.050

**** p<0.01, ** p<0.05, * p<0.1*

Age group: 15-64 years

Mixed-effect Poisson regression model showing the effect of change in unemployment on change in emergency IID admissions per 100,000 adults aged 15-64 years, for English neighbourhoods, 2012–2017

	IRR	St.Err.	t-value	p-value	[95% Conf	Interval]	Sig
Working age population unemployed (%)	1.085	0.001	64.45	<0.001	1.082	1.088	***
2012.year	1.000	.	.	.	.	.	
2013.year	1.008	0.008	1.06	0.287	0.993	1.024	
2014.year	1.152	0.009	17.62	<0.001	1.134	1.170	***
2015.year	1.177	0.010	19.54	<0.001	1.158	1.196	***
2016.year	1.202	0.010	22.21	<0.001	1.183	1.222	***
2017.year	1.198	0.010	21.78	<0.001	1.179	1.218	***
Constant	0.001	0.000	-911.39	<0.001	0.001	0.001	***
Constant	-2.307	0.122	.b	.b	-2.547	-2.067	

Mean dependent var	1.144	SD dependent var	1.228
Number of obs	196974.000	Chi-square	6075404.590
Prob > chi2	0.000	Akaike crit. (AIC)	550137.825

**** p<0.01, ** p<0.05, * p<0.1*

Multivariable mixed-effect Poisson regression model for emergency IID admissions per 100,000 adults aged 15–64 years, for English neighbourhoods, 2012–2017

	IRR	St.Err.	t-value	p-value	[95% Conf	Interval]	Sig
Working age population unemployed (%)	1.024	0.002	14.85	<0.001	1.021	1.028	***
Prevalence long-term health problems, adults aged 15-64 years (%)	1.041	0.001	52.32	<0.001	1.039	1.043	***
Population who would describe their experience of making a GP/nurse appointment as poor (%)	1.004	0.000	8.23	<0.001	1.003	1.005	***
GPs per 1000 population	1.106	0.028	3.98	<0.001	1.053	1.163	***
Travelling distance to hospital (km)	0.994	0.001	-9.87	<0.001	0.993	0.995	***
Ethnic group: Black (%)	1.000	0.000	0.28	0.778	0.999	1.001	
Ethnic group: Chinese (%)	0.987	0.002	-5.83	<0.001	0.983	0.991	***
Ethnic group: Bangladeshi (%)	0.996	0.001	-4.79	<0.001	0.995	0.998	***
Ethnic group: Indian (%)	1.003	0.000	7.24	<0.001	1.002	1.004	***
Ethnic group: Pakistani (%)	1.003	0.000	8.70	<0.001	1.003	1.004	***
2012.year	1.000	.	.	.	.	.	
2013.year	0.971	0.008	-3.82	<0.001	0.956	0.986	***
2014.year	1.028	0.009	3.31	0.001	1.011	1.045	***
2015.year	1.019	0.009	2.12	0.034	1.001	1.036	**
2016.year	1.043	0.009	4.80	<0.001	1.025	1.061	***
2017.year	1.045	0.009	5.04	<0.001	1.027	1.064	***
Constant	0.001	0.000	-415.97	<0.001	0.001	0.001	***
Constant	-2.558	0.144	.b	.b	-2.840	-2.277	

Mean dependent var	1.144	SD dependent var	1.228
Number of obs	196974.000	Chi-square	6677106.012
Prob > chi2	0.000	Akaike crit. (AIC)	546535.025

**** p<0.01, ** p<0.05, * p<0.1*

Age group: 65+ years

Mixed-effect Poisson regression model showing the effect of change in unemployment on change in emergency IID admissions per 100,000 adults aged 65+ years, for English neighbourhoods, 2012–2017

	IRR	St.Err.	t-value	p-value	[95% Conf	Interval]	Sig
Working age population unemployed (%)	1.090	0.002	58.06	<0.001	1.086	1.093	***
2012.year	1.000	.	.	.	.	.	
2013.year	0.982	0.008	-2.32	0.020	0.968	0.997	**
2014.year	1.046	0.009	5.47	<0.001	1.029	1.063	***
2015.year	1.044	0.009	5.03	<0.001	1.026	1.061	***
2016.year	1.001	0.009	0.14	0.886	0.985	1.018	
2017.year	1.006	0.009	0.66	0.509	0.989	1.022	
Constant	0.003	0.000	-688.50	<0.001	0.003	0.003	***
Constant	-2.069	0.112	.b	.b	-2.288	-1.849	

Mean dependent var	1.131	SD dependent var	1.268
Number of obs	196974.000	Chi-square	3465333.121
Prob > chi2	0.000	Akaike crit. (AIC)	545314.139

*** *p<0.01, ** p<0.05, * p<0.1*

Multivariable mixed-effect Poisson regression model for emergency IID admissions per 100,000 adults aged 65+ years, for English neighbourhoods, 2012–2017

	IRR	St.Err.	t-value	p-value	[95% Conf	Interval]	Sig
Working age population unemployed (%)	1.040	0.002	22.11	<0.001	1.036	1.043	***
Prevalence long-term health problems, adults aged 65+ years (%)	1.014	0.000	37.49	<0.001	1.013	1.015	***
Population who would describe their experience of making a GP/nurse appointment as poor (%)	1.005	0.001	8.67	<0.001	1.004	1.006	***
GPs per 1000 population	1.015	0.029	0.52	0.603	0.959	1.074	
Travelling distance to hospital (km)	0.990	0.001	-16.19	<0.001	0.989	0.991	***
Ethnic group: Black (%)	1.003	0.001	5.60	<0.001	1.002	1.004	***
Ethnic group: Chinese (%)	1.025	0.003	8.12	<0.001	1.019	1.031	***
Ethnic group: Bangladeshi (%)	1.001	0.001	1.04	0.298	0.999	1.003	
Ethnic group: Indian (%)	1.000	0.001	0.69	0.493	0.999	1.001	
Ethnic group: Pakistani (%)	1.003	0.001	5.53	<0.001	1.002	1.004	***
2012.year	1.000	.	.	.	.	.	
2013.year	0.952	0.007	-6.29	<0.001	0.938	0.967	***
2014.year	0.959	0.008	-4.99	<0.001	0.943	0.975	***
2015.year	0.935	0.008	-7.59	<0.001	0.919	0.951	***
2016.year	0.899	0.008	-11.99	<0.001	0.883	0.914	***
2017.year	0.905	0.008	-11.19	<0.001	0.890	0.921	***
Constant	0.002	0.000	-248.91	<0.001	0.002	0.002	***
Constant	-2.247	0.127	.b	.b	-2.496	-1.997	

Mean dependent var	1.131	SD dependent var	1.268
Number of obs	196974.000	Chi-square	3846351.406
Prob > chi2	0.000	Akaike crit. (AIC)	542315.876

**** p<0.01, ** p<0.05, * p<0.1*

Robustness tests - fixed-effect models

Fixed-effect Poisson regression model showing the effect of change in unemployment on change in emergency IID admissions per 100,000 children aged 0-14 years, for English neighbourhoods, 2005–2017

	IRR	St.Err.	t-value	p-value	[95% Conf	Interval]	Sig
Working age population unemployed (%)	1.017	0.002	9.44	<0.001	1.013	1.021	***
2005.year	1.000	.	.	.	.	.	
2006.year	1.118	0.008	14.99	<0.001	1.102	1.135	***
2007.year	1.111	0.008	13.89	<0.001	1.095	1.128	***
2008.year	1.114	0.009	14.08	<0.001	1.097	1.130	***
2009.year	1.106	0.009	12.02	<0.001	1.088	1.124	***
2010.year	1.140	0.009	16.41	<0.001	1.122	1.158	***
2011.year	1.046	0.009	5.35	<0.001	1.029	1.064	***
2012.year	1.092	0.009	10.38	<0.001	1.074	1.110	***
2013.year	1.105	0.009	12.35	<0.001	1.088	1.123	***
2014.year	0.846	0.007	-20.38	<0.001	0.832	0.859	***
2015.year	0.846	0.007	-20.10	<0.001	0.832	0.860	***
2016.year	0.846	0.007	-19.90	<0.001	0.833	0.861	***
2017.year	0.829	0.007	-22.24	<0.001	0.816	0.843	***

Mean dependent var	1.327	SD dependent var	1.585
Number of obs	426153.000	Chi-square	6231.219
Prob > chi2	0.000	Akaike crit. (AIC)	1022964.089

**** p<0.01, ** p<0.05, * p<0.1*

Fixed-effect Poisson regression model showing the effect of change in unemployment on change in emergency IID admissions per 100,000 adults aged 15-64 years, for English neighbourhoods, 2005–2017

	IRR	St.Err.	t-value	p-value	[95% Conf	Interval]	Sig
Working age population unemployed (%)	1.018	0.002	9.34	<0.001	1.014	1.022	***
2005.year	1.000	.	.	.	.	.	
2006.year	1.115	0.010	12.65	<0.001	1.096	1.134	***
2007.year	1.145	0.010	15.66	<0.001	1.126	1.165	***
2008.year	1.103	0.010	11.38	<0.001	1.085	1.122	***
2009.year	1.122	0.011	12.29	<0.001	1.102	1.143	***
2010.year	1.219	0.011	22.14	<0.001	1.198	1.240	***
2011.year	1.164	0.011	16.53	<0.001	1.143	1.185	***
2012.year	1.259	0.011	25.29	<0.001	1.237	1.282	***
2013.year	1.229	0.011	23.29	<0.001	1.208	1.250	***
2014.year	1.298	0.011	31.10	<0.001	1.277	1.320	***
2015.year	1.284	0.011	29.41	<0.001	1.263	1.306	***
2016.year	1.310	0.011	32.03	<0.001	1.288	1.332	***
2017.year	1.315	0.011	32.22	<0.001	1.293	1.337	***

Mean dependent var	1.059	SD dependent var	1.178
Number of obs	426569.000	Chi-square	2146.640
Prob > chi2	0.000	Akaike crit. (AIC)	948657.908

**** p<0.01, ** p<0.05, * p<0.1*

Fixed-effect Poisson regression model showing the effect of change in unemployment on change in emergency IID admissions per 100,000 adults aged 65+ years, for English neighbourhoods, 2005–2017

	IRR	St.Err.	t-value	p-value	[95% Conf	Interval]	Sig
Working age population unemployed (%)	1.010	0.002	4.88	<0.001	1.006	1.014	***
2005.year	1.000	.	.	.	.	.	
2006.year	1.178	0.010	18.86	<0.001	1.158	1.198	***
2007.year	1.259	0.011	26.31	<0.001	1.238	1.281	***
2008.year	1.188	0.010	19.62	<0.001	1.168	1.208	***
2009.year	1.177	0.011	17.10	<0.001	1.155	1.199	***
2010.year	1.260	0.011	25.82	<0.001	1.239	1.283	***
2011.year	1.185	0.011	18.26	<0.001	1.164	1.207	***
2012.year	1.253	0.012	24.49	<0.001	1.230	1.275	***
2013.year	1.187	0.011	19.30	<0.001	1.167	1.208	***
2014.year	1.165	0.010	17.72	<0.001	1.146	1.185	***
2015.year	1.127	0.010	13.67	<0.001	1.108	1.146	***
2016.year	1.082	0.009	8.97	<0.001	1.063	1.100	***
2017.year	1.096	0.009	10.55	<0.001	1.077	1.115	***

Mean dependent var	1.084	SD dependent var	1.259
Number of obs	424281.000	Chi-square	1665.581
Prob > chi2	0.000	Akaike crit. (AIC)	943602.473

**** p<0.01, ** p<0.05, * p<0.1*

Robustness tests – negative binomial models

Multivariable mixed-effect negative binomial regression model for emergency IID admissions per 100,000 children aged 0–14 years, for English neighbourhoods, 2012–2017

	IRR	St.Err.	t-value	p-value	[95% Conf	Interval]	Sig
Working age population unemployed (%)	1.065	0.002	42.12	<0.001	1.062	1.069	***
Prevalence long-term health problems, children aged 0-14 years (%)	1.041	0.002	17.52	<0.001	1.036	1.046	***
Population who would describe their experience of making a GP/nurse appointment as poor (%)	1.002	0.001	3.79	<0.001	1.001	1.003	***
GPs per 1000 population	0.924	0.027	-2.65	0.008	0.872	0.980	***
Travelling distance to hospital (km)	0.996	0.001	-5.81	<0.001	0.994	0.997	***
Ethnic group: Black (%)	0.984	0.001	-27.80	<0.001	0.983	0.985	***
Ethnic group: Chinese (%)	0.976	0.003	-6.84	<0.001	0.969	0.983	***
Ethnic group: Bangladeshi (%)	0.991	0.001	-8.64	<0.001	0.989	0.993	***
Ethnic group: Indian (%)	0.998	0.001	-2.64	0.008	0.997	1.000	***
Ethnic group: Pakistani (%)	1.009	0.001	17.62	<0.001	1.008	1.010	***
2012.year	1.000	.	.	.	.	.	
2013.year	1.032	0.007	4.46	<0.001	1.018	1.047	***
2014.year	0.838	0.007	-21.28	<0.001	0.824	0.852	***
2015.year	0.857	0.007	-17.70	<0.001	0.842	0.872	***
2016.year	0.857	0.007	-17.79	<0.001	0.842	0.871	***
2017.year	0.834	0.007	-20.72	<0.001	0.820	0.848	***
Constant	0.022	0.001	-117.34	<0.001	0.021	0.023	***
Constant	3.479	0.023	.b	.b	3.434	3.524	
Constant	1.668	0.016	.b	.b	1.638	1.699	

Mean dependent var	1.249	SD dependent var	1.534
Number of obs	196974.000	Chi-square	8817.042
Prob > chi2	0.000	Akaike crit. (AIC)	561066.055

**** p<0.01, ** p<0.05, * p<0.1*

Multivariable mixed-effect negative binomial regression model for emergency IID admissions per 100,000 adults aged 15–64 years, for English neighbourhoods, 2012–2017

	IRR	St.Err.	t-value	p-value	[95% Conf	Interval]	Sig
Working age population unemployed (%)	1.025	0.002	15.28	<0.001	1.021	1.028	***
Prevalence long-term health problems, adults aged 15-64 years (%)	1.041	0.001	53.68	<0.001	1.039	1.042	***
Population who would describe their experience of making a GP/nurse appointment as poor (%)	1.004	0.000	8.59	<0.001	1.003	1.005	***
GPs per 1000 population	1.115	0.028	4.36	<0.001	1.062	1.171	***
Travelling distance to hospital (km)	0.994	0.001	-9.80	<0.001	0.993	0.995	***
Ethnic group: Black (%)	1.000	0.000	0.18	0.857	0.999	1.001	
Ethnic group: Chinese (%)	0.987	0.002	-5.80	<0.001	0.983	0.992	***
Ethnic group: Bangladeshi (%)	0.996	0.001	-4.90	<0.001	0.995	0.998	***
Ethnic group: Indian (%)	1.003	0.000	6.95	<0.001	1.002	1.004	***
Ethnic group: Pakistani (%)	1.003	0.000	8.18	<0.001	1.002	1.004	***
2012.year	1.000	.	.	.	.	.	
2013.year	0.970	0.008	-3.94	<0.001	0.955	0.985	***
2014.year	1.027	0.009	3.22	0.001	1.010	1.044	***
2015.year	1.018	0.009	2.04	0.041	1.001	1.036	**
2016.year	1.042	0.009	4.79	<0.001	1.025	1.060	***
2017.year	1.044	0.009	4.96	<0.001	1.026	1.062	***
Constant	0.006	0.000	-118.24	<0.001	0.005	0.006	***
Constant	5.195	0.039	.b	.b	5.118	5.271	
Constant	2.859	0.031	.b	.b	2.798	2.919	

Mean dependent var	1.144	SD dependent var	1.228
Number of obs	196974.000	Chi-square	9677.704
Prob > chi2	0.000	Akaike crit. (AIC)	545779.049

**** p<0.01, ** p<0.05, * p<0.1*

Multivariable mixed-effect negative binomial regression model for emergency IID admissions per 100,000 adults aged 65+ years, for English neighbourhoods, 2012–2017

	IRR	St.Err.	t-value	p-value	[95% Conf	Interval]	Sig
Working age population unemployed (%)	1.039	0.002	24.52	<0.001	1.036	1.042	***
Prevalence long-term health problems, adults aged 65+ years (%)	1.014	0.000	42.93	<0.001	1.014	1.015	***
Population who would describe their experience of making a GP/nurse appointment as poor (%)	1.005	0.001	9.10	<0.001	1.004	1.006	***
GPs per 1000 population	1.015	0.027	0.56	0.575	0.964	1.068	
Travelling distance to hospital (km)	0.990	0.001	-16.22	<0.001	0.989	0.991	***
Ethnic group: Black (%)	1.003	0.001	5.70	<0.001	1.002	1.004	***
Ethnic group: Chinese (%)	1.025	0.003	8.32	<0.001	1.019	1.031	***
Ethnic group: Bangladeshi (%)	1.001	0.001	1.00	0.316	0.999	1.003	
Ethnic group: Indian (%)	1.000	0.001	0.88	0.380	0.999	1.001	
Ethnic group: Pakistani (%)	1.003	0.001	5.53	<0.001	1.002	1.004	***
2012.year	1.000	.	.	.	.	.	
2013.year	0.952	0.007	-6.26	<0.001	0.938	0.967	***
2014.year	0.958	0.008	-5.19	<0.001	0.942	0.974	***
2015.year	0.933	0.008	-8.01	<0.001	0.917	0.949	***
2016.year	0.898	0.008	-12.39	<0.001	0.883	0.914	***
2017.year	0.905	0.008	-11.59	<0.001	0.889	0.920	***
Constant	0.013	0.001	-113.97	<0.001	0.012	0.014	***
Constant	4.609	0.030	.b	.b	4.549	4.668	
Constant	2.591	0.026	.b	.b	2.540	2.642	

Mean dependent var	1.131	SD dependent var	1.268
Number of obs	196974.000	Chi-square	9165.980
Prob > chi2	0.000	Akaike crit. (AIC)	540877.432

*** p<0.01, ** p<0.05, * p<0.1*

Robustness tests – using different definition of IID excluding ICD-10 codes: K52.9 and A09.9 (gastroenteritis and colitis of unspecified origin)

Multivariable mixed-effect Poisson regression model for emergency IID admissions per 100,000 children aged 0–14 years, for English neighbourhoods, 2012–2017

	IRR	St.Err.	t-value	p-value	[95% Conf	Interval]	Sig
Working age population unemployed (%)	1.078	0.002	36.27	<0.001	1.074	1.082	***
Prevalence long-term health problems, children aged 0-14 years (%)	1.038	0.003	13.26	<0.001	1.033	1.044	***
Population who would describe their experience of making a GP/nurse appointment as poor (%)	1.002	0.001	2.20	0.028	1.000	1.003	**
GPs per 1000 population	0.970	0.036	-0.82	0.410	0.901	1.043	
Travelling distance to hospital (km)	0.995	0.001	-5.10	<0.001	0.994	0.997	***
Ethnic group: Black (%)	0.973	0.001	-32.30	<0.001	0.971	0.975	***
Ethnic group: Chinese (%)	0.954	0.005	-8.37	<0.001	0.944	0.965	***
Ethnic group: Bangladeshi (%)	0.989	0.002	-6.95	<0.001	0.986	0.992	***
Ethnic group: Indian (%)	0.997	0.001	-3.59	<0.001	0.996	0.999	***
Ethnic group: Pakistani (%)	1.009	0.001	14.37	<0.001	1.008	1.011	***
2012.year	1.000	.	.	.	.	.	
2013.year	1.035	0.009	3.79	<0.001	1.017	1.054	***
2014.year	0.832	0.009	-16.91	<0.001	0.815	0.850	***
2015.year	0.871	0.010	-12.30	<0.001	0.852	0.890	***
2016.year	0.872	0.010	-12.07	<0.001	0.852	0.891	***
2017.year	0.844	0.010	-14.81	<0.001	0.826	0.863	***
Constant	0.002	0.000	-230.66	<0.001	0.002	0.002	***
Constant	-1.069	0.083	.b	.b	-1.231	-0.906	

Mean dependent var	0.749	SD dependent var	1.121
Number of obs	196932.000	Chi-square	1988064.899
Prob > chi2	0.000	Akaike crit. (AIC)	439122.154

*** p<0.01, ** p<0.05, * p<0.1*

Multivariable mixed-effect Poisson regression model for emergency IID admissions per 100,000 adults aged 15–64 years, for English neighbourhoods, 2012–2017

	IRR	St.Err.	t-value	p-value	[95% Conf	Interval]	Sig
Working age population unemployed (%)	1.024	0.003	8.98	<0.001	1.019	1.029	***
Prevalence long-term health problems, adults aged 15-64 years (%)	1.038	0.001	31.13	<0.001	1.036	1.041	***
Population who would describe their experience of making a GP/nurse appointment as poor (%)	1.003	0.001	3.72	<0.001	1.001	1.005	***
GPs per 1000 population	1.387	0.056	8.05	<0.001	1.281	1.502	***
Travelling distance to hospital (km)	0.995	0.001	-5.24	<0.001	0.993	0.997	***
Ethnic group: Black (%)	0.994	0.001	-8.30	<0.001	0.993	0.996	***
Ethnic group: Chinese (%)	0.994	0.003	-1.77	0.076	0.987	1.001	*
Ethnic group: Bangladeshi (%)	0.998	0.001	-1.57	0.116	0.995	1.001	
Ethnic group: Indian (%)	1.003	0.001	4.99	<0.001	1.002	1.005	***
Ethnic group: Pakistani (%)	1.006	0.001	10.57	<0.001	1.005	1.007	***
2012.year	1.000	.	.	.	.	.	
2013.year	0.978	0.014	-1.54	0.123	0.952	1.006	
2014.year	1.037	0.015	2.49	0.013	1.008	1.068	**
2015.year	1.070	0.017	4.38	<0.001	1.038	1.103	***
2016.year	1.166	0.018	10.12	<0.001	1.132	1.201	***
2017.year	1.172	0.018	10.41	<0.001	1.137	1.208	***
Constant	0.000	0.000	-302.90	<0.001	0.000	0.000	***
Constant	-2.194	0.259	.b	.b	-2.702	-1.687	

Mean dependent var	0.351	SD dependent var	0.630
Number of obs	196932.000	Chi-square	3521975.686
Prob > chi2	0.000	Akaike crit. (AIC)	296108.573

*** *p<0.01*, ** *p<0.05*, * *p<0.1*

Multivariable mixed-effect Poisson regression model for emergency IID admissions per 100,000 adults aged 65+ years, for English neighbourhoods, 2012–2017

	IRR	St.Err.	t-value	p-value	[95% Conf	Interval]	Sig
Working age population unemployed (%)	1.043	0.003	15.18	<0.001	1.038	1.049	***
Prevalence long-term health problems, adults aged 65+ years (%)	1.015	0.001	23.87	<0.001	1.013	1.016	***
Population who would describe their experience of making a GP/nurse appointment as poor (%)	1.003	0.001	3.24	0.001	1.001	1.005	***
GPs per 1000 population	1.252	0.055	5.11	<0.001	1.149	1.365	***
Travelling distance to hospital (km)	0.989	0.001	-10.63	<0.001	0.987	0.991	***
Ethnic group: Black (%)	0.997	0.001	-2.63	0.009	0.996	0.999	***
Ethnic group: Chinese (%)	1.028	0.005	5.74	<0.001	1.018	1.037	***
Ethnic group: Bangladeshi (%)	1.001	0.002	0.40	0.693	0.997	1.005	
Ethnic group: Indian (%)	0.998	0.001	-1.79	0.074	0.997	1.000	*
Ethnic group: Pakistani (%)	1.005	0.001	6.19	<0.001	1.004	1.007	***
2012.year	1.000	.	.	.	.	.	
2013.year	0.926	0.014	-5.22	<0.001	0.900	0.953	***
2014.year	0.976	0.015	-1.53	0.126	0.947	1.007	
2015.year	1.012	0.016	0.73	0.465	0.980	1.044	
2016.year	0.995	0.016	-0.34	0.738	0.964	1.026	
2017.year	1.029	0.016	1.76	0.078	0.997	1.061	*
Constant	0.000	0.000	-193.31	<0.001	0.000	0.000	***
Constant	-1.680	0.195	.b	.b	-2.063	-1.297	

Mean dependent var	0.322	SD dependent var	0.625
Number of obs	196932.000	Chi-square	2080123.421
Prob > chi2	0.000	Akaike crit. (AIC)	280369.703

**** p<0.01, ** p<0.05, * p<0.1*
